# Evolutionary Loss of Activity in De-Ubiquitylating Enzymes of the OTU Family

**DOI:** 10.1371/journal.pone.0143227

**Published:** 2015-11-20

**Authors:** Marcell Louis, Kay Hofmann, Meike Broemer

**Affiliations:** 1 German Center for Neurodegenerative Diseases (DZNE), Bonn, c/o LiMeS, Carl-Troll-Str. 31, 53115, Bonn, Germany; 2 University of Cologne, Institute for Genetics, Zülpicher Str. 47a, 50674, Cologne, Germany; Karolinska Institutet, SWEDEN

## Abstract

Understanding function and specificity of de-ubiquitylating enzymes (DUBs) is a major goal of current research, since DUBs are key regulators of ubiquitylation events and have been shown to be mutated in human diseases. Most DUBs are cysteine proteases, relying on a catalytic triad of cysteine, histidine and aspartate to cleave the isopeptide bond between two ubiquitin units in a poly-ubiquitin chain. We have discovered that the two *Drosophila melanogaster* homologues of human OTUD4, CG3251 and Otu, contain a serine instead of a cysteine in the catalytic OTU (ovarian tumor) domain. DUBs that are serine proteases instead of cysteine- or metallo-proteases have not been described. In line with this, neither CG3251 nor Otu protein were active to cleave ubiquitin chains. Re-introduction of a cysteine in the catalytic center did not render the enzymes active, indicating that further critical features for ubiquitin binding or cleavage have been lost in these proteins. Sequence analysis of OTUD4 homologues from various other species showed that within this OTU subfamily, loss of the catalytic cysteine has occurred frequently in presumably independent events, as well as gene duplications or triplications, suggesting DUB-independent functions of OTUD4 proteins. Using an *in vivo* RNAi approach, we show that CG3251 might function in the regulation of Inhibitor of Apoptosis (IAP)-antagonist-induced apoptosis, presumably in a DUB-independent manner.

## Introduction

Posttranslational modification of proteins with mono- or poly-ubiquitin is a highly versatile tool to control for example protein stability, protein localization or signaling processes. Ubiquitin gets covalently linked to lysine residues of the target protein or to lysines within another ubiquitin molecule, which leads to the formation of poly-ubiquitin chains. These chains can be linked through seven different internal lysines. Additionally, linkages can be formed through the alpha-amino group of ubiquitin. Different chain types are recognized by proteins with specialized ubiquitin-binding domains (UBD), which mediate downstream events such as proteasomal degradation or formation of protein complexes [1].

An important regulatory mechanism is the removal of ubiquitin chains by de-ubiquitylating enzymes (DUBs). Five different classes of DUBs have been described, based on their type of protease domain: Ubiquitin-specific proteases (USPs), Ubiquitin-COOH-terminal hydrolases (UCH), Ovarian Tumor- (OTU) type DUBs, Josephins and JAB1/MPN/MOV34 (JAMM)-metallo-proteases [2]. With exception of the JAMM-metallo-proteases, all known de-ubiquitylating enzymes are cysteine (C, Cys) proteases and rely on a critical cysteine in their protease domain. Usually, this cysteine forms a catalytic triad together with a histidine and aspartate to catalyze the cleavage of the isopeptide bond between two ubiquitin units [3–5].

The importance of proper regulation of protein ubiquitylation is reflected by the identification of mutations in DUB genes in diseases such as cancer and neurodegeneration [2]. Therefore, understanding function and regulation of the approximately 90 human DUBs has a high priority and model organisms such as *Drosophila melanogaster* are a valuable tool to shed light on DUB biology.

We have analyzed two *Drosophila melanogaster* DUBs of the OTU-family. The OTU family of DUBs is particularly interesting because many of these DUBs display specificity for one or several types of ubiquitin chain linkages. Of the 18 genes with OTU domains in the human genome, 16 contain an intact catalytic triad. In contrast, HIN1L is a pseudogene and FAM105A does not have an intact catalytic triad [6]. The *Drosophila melanogaster* genome encodes for seven OTU-domain containing proteins (M.B., K.H. and Pascal Meier, unpublished data), of which only Trabid and Otu (ovarian tumor) have been characterized regarding their biological role [7–10]. Our analysis revealed that two of the potential OTU DUBs from *Drosophila melanogaster*, CG3251 and Otu (shared homologues of human OTUD4) carry a serine (S, Ser) instead of the central cysteine in the catalytic triad. While a high number of serine proteases exist [11, 12], ubiquitin-specific serine proteases have not been described. We demonstrate here that CG3251 and Otu both lack DUB activity *in vitro*. Furthermore, our bioinformatical analysis of the OTUD4 family proteins in other species revealed that loss of the catalytic cysteine seems to have occurred frequently in independent events. Taken together, our data suggest that the two *Drosophila melanogaster* OTUD4 homologues exert a DUB-independent function.

## Results

The functional role of many de-ubiquitylating enzymes has not been elucidated satisfyingly and with this work we aim to shed light on the role of the previously uncharacterized *Drosophila melanogaster* gene *CG3251*, a homologue of the mammalian DUB OTUD4.

**Fig 1:**
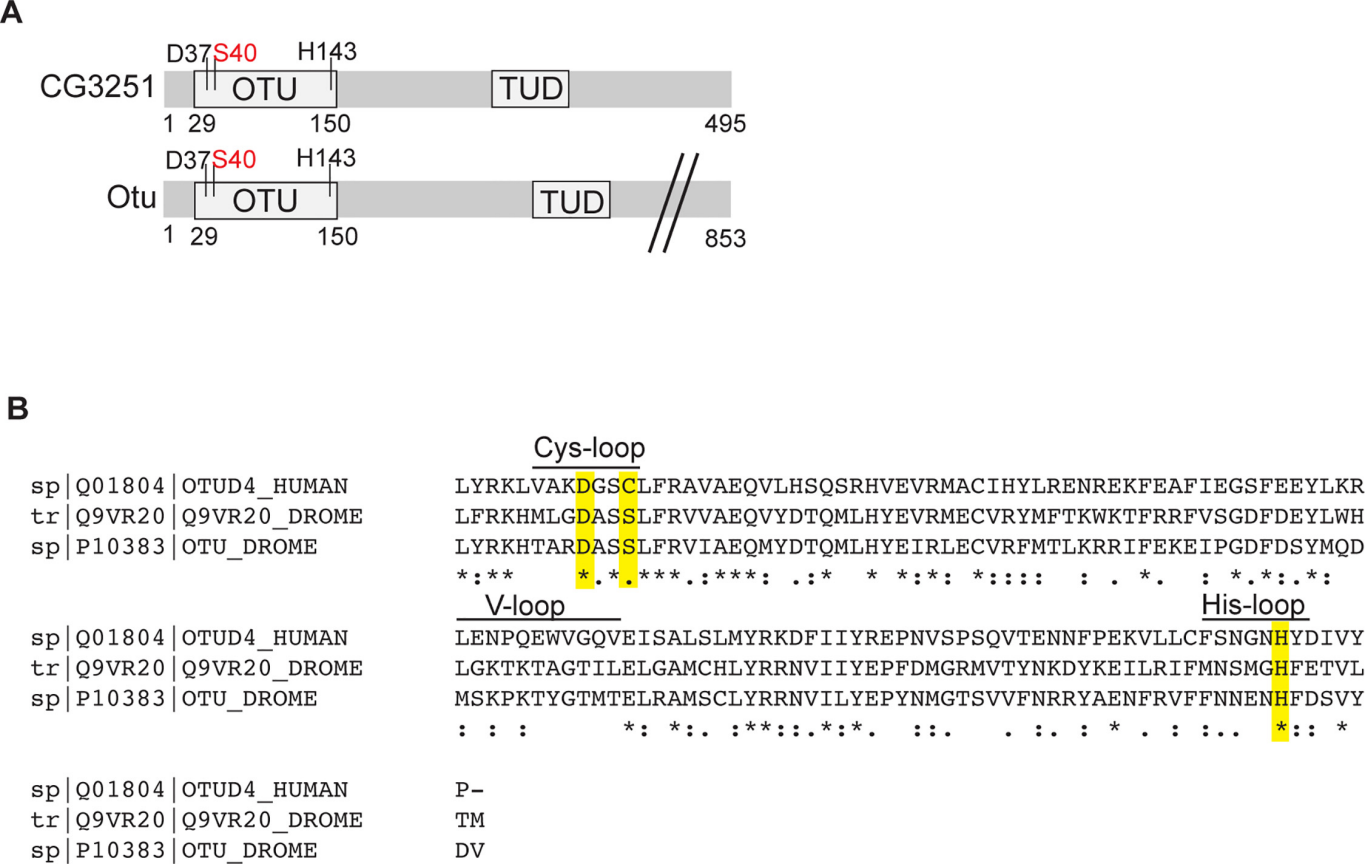
(A) Schematic domain structure of the *Drosophila* proteins CG3251 and Otu. CG3251 is a 495 amino acid (aa) protein, with an OTU domain (position 29–150) and a Tudor domain (“TUD”, 296–361). The residues of the predicted catalytic triad are D37, S40 and H143. The Otu protein carries an OTU domain with identical features and also a Tudor domain (336–396). The full-length protein consists of 853 amino acids (not drawn to scale). The presence of Ser instead of Cys in the catalytic triad (S40) is highlighted by red colour. (B) Alignment of protein sequence of OTU domains of OTUD4 (human), aa 34–155, CG3251 (Q9VR20), aa 29–150 and Otu, aa 29–150. Fully conserved residues are marked by “*”, “:” denotes strongly similar residues and “.” weakly similar residues, according to Clustal Omega analysis [15]. The catalytic triad is highlighted in yellow and conserved areas required for catalytic activity are denoted as Cys-, His- and V(ariable)-loop, respectively.

*CG3251* encodes for a protein of 495 amino acids with an OTU domain, making it a member of the OTU family of de-ubiquitiylating enzymes (Fig 1A). Additionally, the protein contains a putative Tudor domain, which in other cases has been shown to interact with RNA [13] or methylated Histones [14].

Usually, OTU-type DUBs process ubiquitin chains using three conserved residues in their OTU domain to orient and cleave ubiquitin. This catalytic triad typically consists of a central cysteine, in structural proximity with an aspartate (D) and a histidine (H) [3–5]. Analysis of the potential catalytic triad of CG3251 revealed a lack of the catalytic cysteine, which is replaced by serine (S40, Fig 1A and 1B), while D37 and H143 represent the additional two amino acids of the triad. CG3251 and the product of the *Drosophila ovarian tumour* (*otu*) gene, Otu, are considered as shared homologues of the human DUB OTUD4. Alignment of the OTU domains of CG3251 (UniProt accession number Q9VR20), Otu and OTUD4 is shown in Fig 1B and the position of amino acids of the catalytic triad is highlighted in yellow. Interestingly, also Otu does not contain a cysteine but a serine in the catalytic triad, in contrast to OTUD4, which carries a cysteine.

### Cysteine to serine mutation in homologues of OTUD4 is a frequent event throughout evolution

We wondered whether the loss of the catalytic cysteine of CG3251 and Otu is a hallmark of *Drosophila melanogaster* OTUD4 homologues or whether this change in sequence has also occurred in other species. Mammals contain two genes of this family: *OTUD4*, *ALG13* and one pseudogene, *HIN1L*, presumably the products of a gene triplication. All three genes encode for a cysteine in the catalytic triad of the OTU domain but so far only OTUD4 has been shown to have DUB activity *in vitro* [6, 16]. To investigate at what point(s) in evolution the cysteine-to-serine mutation in the catalytic triad occurred, we collected sequences of the CG3251/OTUD4 family from a diverse set of species with completely sequenced genomes. After aligning the sequences with the L-ins-I program of the MAFFT package [17], a neighbor-joining dendrogram [18] was generated and used to remove sequences belonging to other (non-CG3251/OTUD4) subfamilies of the OTU superfamily. The remaining sequences were assessed for their residue choice at the position of the catalytic cysteine.

**Fig 2:**
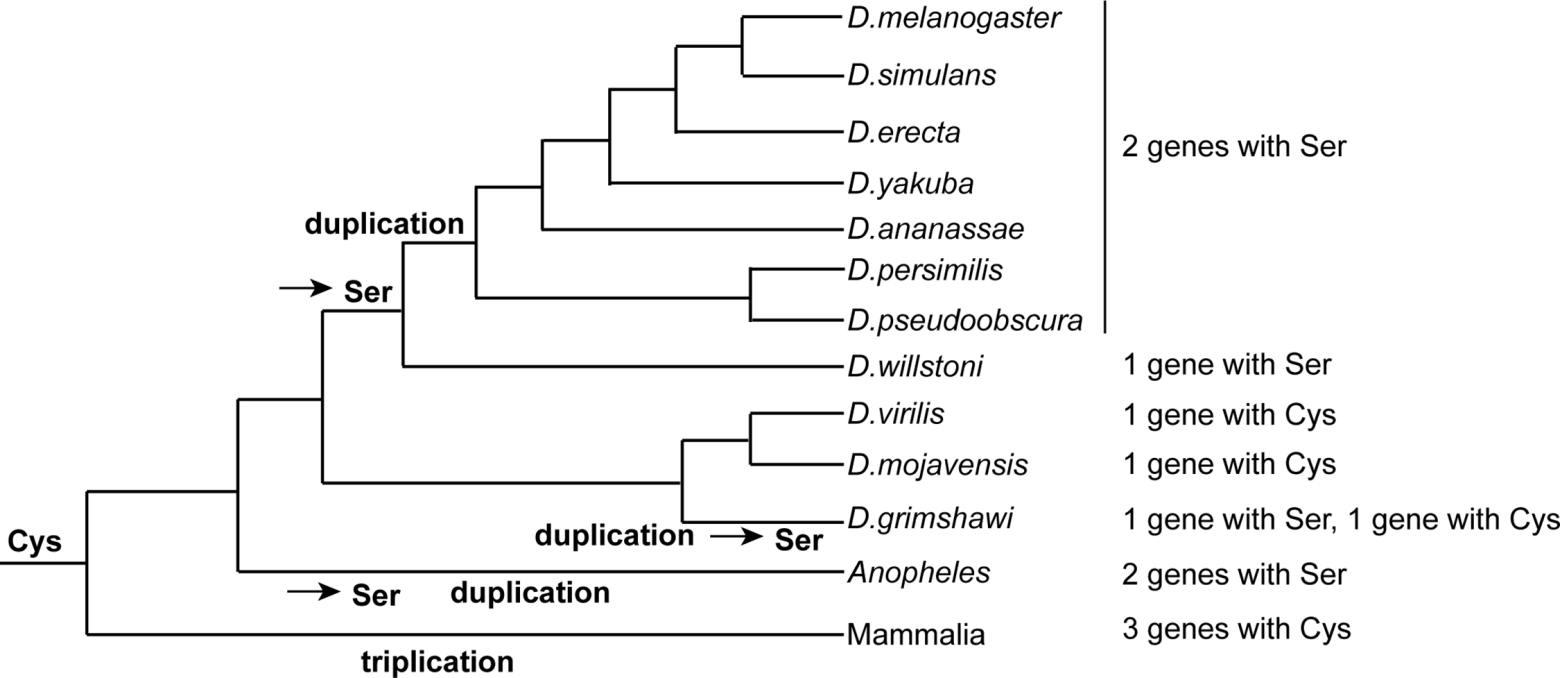
Occurrence of Cys-to-Ser mutations in proteins of the CG3251/OTUD4 family during *Drosophila* evolution. To compare the occurrence of Cys-to-Ser mutations in the catalytic triad of the OTU domain, protein sequences of proteins of the OTUD4 family were analysed as described in the main text. Shown is the most likely order of gene amplifications and independent Cys-to-Ser mutations in relation to *Drosophila* evolution.

Assuming that all members of the CG3251/OTUD4 family have evolved from a common ancestor–most likely a catalytically active protease with an active site cysteine–there is evidence for multiple independent Cys-to-Ser mutations. While mammals and most other animal lineages only encode OTUD4 subfamily members with active site cysteine, several insects possess family members with a Cys-to-Ser mutation. *Drosophila melanogaster* and its closely related sister species including *D*.*erecta*, *D*.*yakuba*, and *D*.*pseudoobscura* all code for two serine-containing OTUD4 family members that arose through a gene duplication after the split from *D*.*willstoni*, which encodes a single serine-containing gene. By contrast, in the earlier branching drosophilids *D*.*virilis* and *D*.*mojavensis*, the corresponding gene has retained the active-site cysteine. In the closely related *D*.*grimshawi*, this gene has been duplicated independently, with one copy showing a cysteine-to-serine mutation. A third independent duplication with Cys-to-Ser mutations happened in the lineage leading to *Anopheles* mosquitos. Fig 2 shows the most likely series of events mapped onto a species tree of *Drosophila* evolution [19].

Other insect lineages like beetles (e.g. *Tribolium castaneum*) or butterflies (e.g. *Danaus plexippus*) generally encode for cysteine-containing OTUD4 proteins. Occasional cysteine-to-serine mutations are also found outside the insect class: while most fish species encode a conventional cysteine-containing OTUD4 homologue, the corresponding protein of the pufferfish *Fugu rubripes* has an active-site serine. Taken together, a range of combinations with single or multiple proteins with either cysteine or serine OTU domains exist in this family. Up to this point, it has remained unclear whether serine-containing OTU DUBs can process ubiquitin chains. The fact that Cys-Ser exchanges have occurred so frequently suggests that even with a serine in the catalytic triad OTU-type DUBs might be active in processing ubiquitin chains *in vivo*.

### CG3251 does not function as a DUB

**Fig 3:**
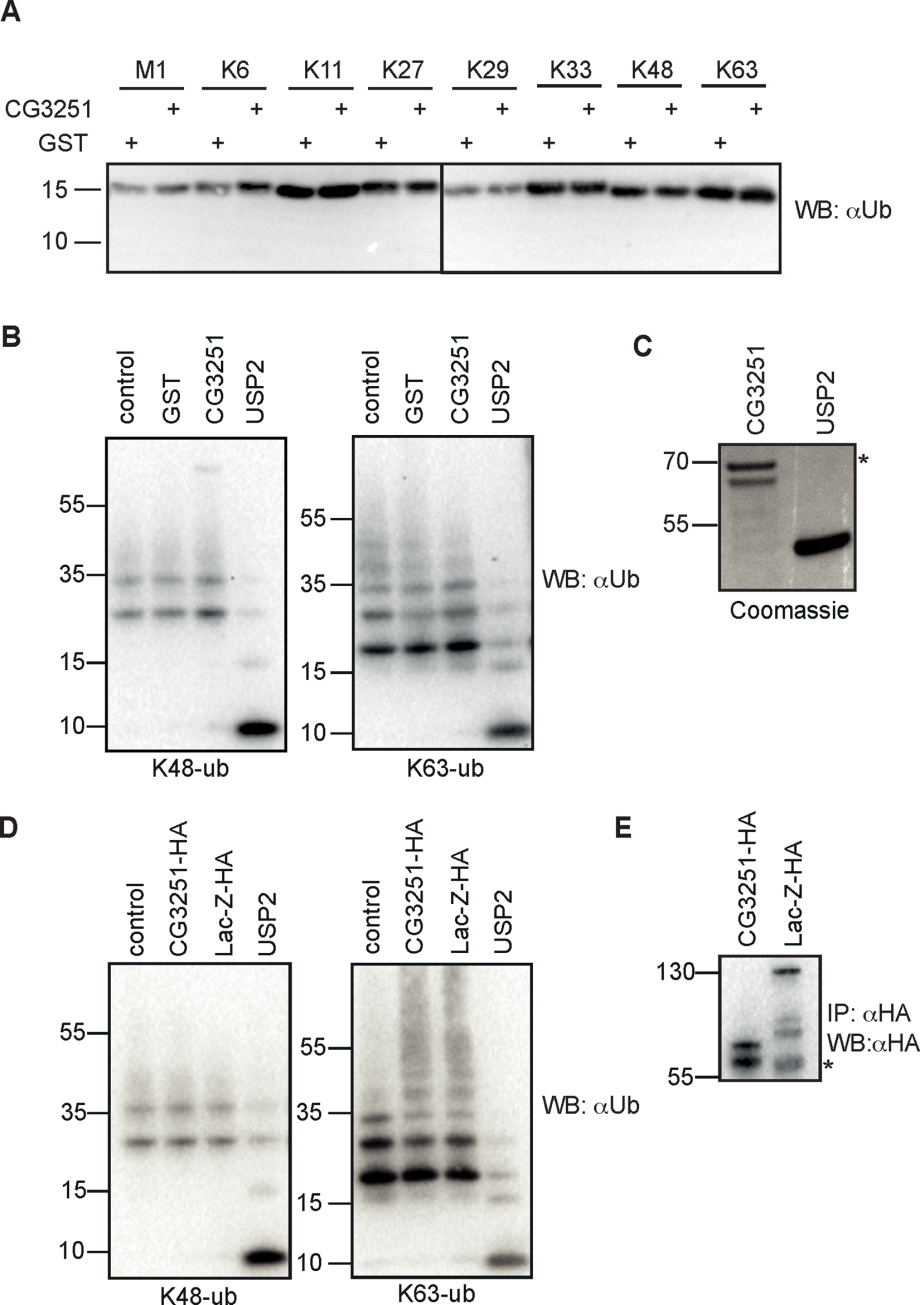
CG3251 does not process ubiquitin chains in vitro. **(A)** Activity test of recombinant CG3251 protein on di-ubiquitin molecules. GST-CG3251 or GST protein as negative control was incubated with di-ubiquitin molecules of the indicated linkage types and cleavage assessed by anti-ubiquitin (Ub) Western Blot. None of the chain types was cut by CG3251. **(B)** Activity test on longer ubiquitin chains. K48- and K63-linked ubiquitin chains (3–7 ubiquitin units) were incubated with buffer only or recombinant proteins (GST, CG3251, USP2) and ubiquitin processing analyzed by WB. **(C)** Coomassie-stained gel to show input levels of CG3251 and USP2, respectively. * denotes a co-purified *E*. *coli* protein in the CG3251 preparation. **(D)** *Drosophila* S2 cells were transfected with expression constructs for HA-tagged CG3251 or HA-lacZ as control. Immunoprecipitated CG3251-HA or lacZ-HA was incubated with ubiquitin chains as in (B). Control sample was incubated with buffer only and recombinant USP2 was used as positive control. **(E)** anti-HA immunoprecipitates as used for cleavage reaction in D). * denotes the IgG heavy chain.

To test for DUB activity of CG3251, we purified CG3251 protein from *E*. *coli* and assessed cleavage of di-ubiquitin molecules and longer K48- and K63- linked ubiquitin chains *in vitro*. Despite extended reaction time of up to 16 h and testing of different buffer conditions (data not shown) we were not able to observe processing of any di-ubiquitin linkage type (M1, K6, K11, K27, K29, K33, K48, K63) (Fig 3A). Furthermore, CG3251 did not cleave longer (3–7) K48- or K63-linked ubiquitin chains (Fig 3B). Input levels of recombinant protein are shown in Fig 3C.

Some DUBs require posttranslational activation steps for activity. To test CG3251 activity from another source we expressed HA-tagged CG3251 in *Drosophila* S2 cells to potentially enable posttranslational modifications. Following immunopurification of HA-CG3251 or HA-lacZ (as unrelated control protein) with anti-HA-affinity resin from cell lysate, reaction buffer with K48- or K63-ubiquitin chains was added to beads and cleavage of ubiquitin chains monitored by anti-ubiquitin Western Blotting (Fig 3D). Successful immunoprecipitation of HA-tagged proteins was controlled by anti-HA Western Blotting (Fig 3E). As with CG3251 from *E*. *coli*, we were not able to detect any DUB activity of CG3251, suggesting that the Cys-to-Ser mutation renders the enzyme inactive for cleavage of ubiquitin chains. Furthermore, CG3251 was not able to interact with a specific probe (HA-Ub-Vinyl sulfone), which covalently and irreversibly reacts with active de-ubiquitylating enzymes, again arguing against catalytic activity of this protein (data not shown).

### Re-mutation of S40 to cysteine does not create active CG3251 enzyme

**Fig 4:**
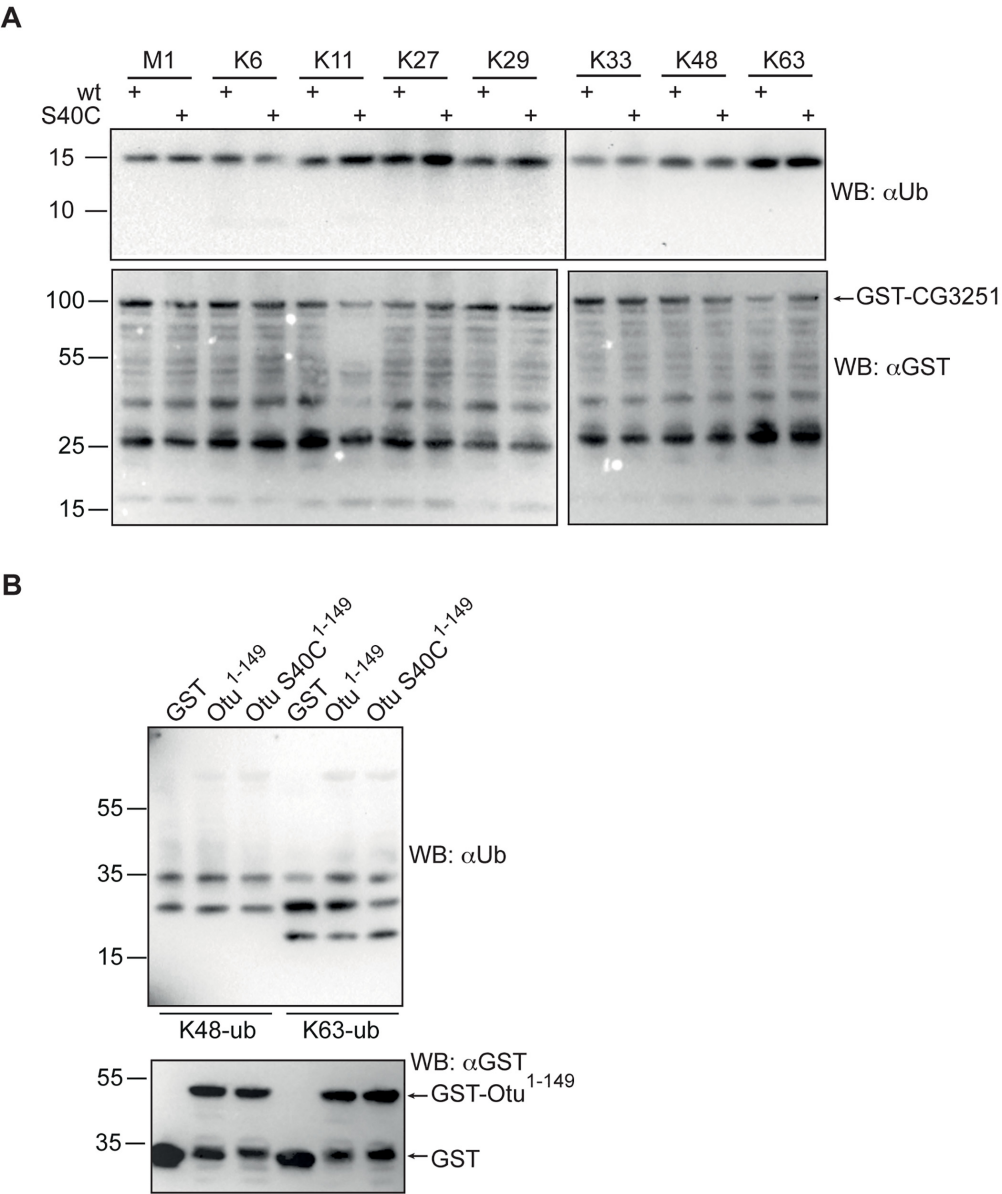
Re-mutation of S40 to cysteine does not restore catalytic activity of CG3251 and Otu protein. **(A)** To test whether mutation of S40 to cysteine restored activity, wild type and S40C-mutated CG3251 protein from *E*.*coli* was incubated with di-ubiquitin molecules of all linkage types and potential ubiquitin cleavage assessed by WB. Cleavage activity was not observed. Input levels of GST-CG3251 or -CG3251 S40C are shown by anti-GST WB. Full-length GST-CG3251 is indicated by arrow, while also multiple truncated forms are present in the protein sample. **(B)** Otu and Otu S40C are inactive DUBs. An NH_2_-terminal fragment encoding the OTU domain of the Otu protein (aa 1–149) was expressed in *E*. *coli* as GST-fusion protein. Wild type and an S40C-mutated form were used. Cleavage of K48- or K63-linked ubiquitin chains was determined by anti-ubiquitin WB and the input of recombinant proteins was controlled by anti-GST WB.

If the Cys-to-Ser exchange in the catalytic triad was the only cause for enzymatic inactivity of CG3251, a mutation to cysteine in this position should re-establish DUB activity. To test this, we performed site-directed mutagenesis and compared activity of wild type or S40C GST-CG3251 protein, expressed in *E*. *coli*, in its ability to process di-ubiquitin molecules. Unexpectedly, also the S40C CG3251 did not show activity *in vitro* under the conditions tested (Fig 4A). This result suggests that further features apart from the cysteine in the catalytic triad of CG3251 have been lost, contributing to inactivation of DUB activity. Parallel experiments were also performed for Otu, which also carries a serine instead of a cysteine (Fig 1A and 1B). The N-terminal part (aa 1–149), comprising the OTU domain was purified as GST-fusion protein from bacteria. Of note, isolated OTU domains of active DUBs carry activity *in vitro*. The protein was tested in its wild type version and with S40C substitution for cleavage of ubiquitin chains. However, the Otu protein also did not cleave ubiquitin chains *in vitro* and the re-introduction of a Cys in the catalytic triad did not re-establish DUB activity to process K48- or K63-linked ubiquitin chains (Fig 4B).

Taken together, our results show that CG3251 and Otu are not active as de-ubiquitylating enzymes. While the cysteine-to-serine exchange in the catalytic triad might contribute to loss of activity for this class of OTU proteins, it does not seem to be the only sequence or structure modification that has occurred to make CG3251 and Otu inactive as a DUB.

### CG3251 is involved in the regulation of apoptosis in vivo

**Fig 5:**
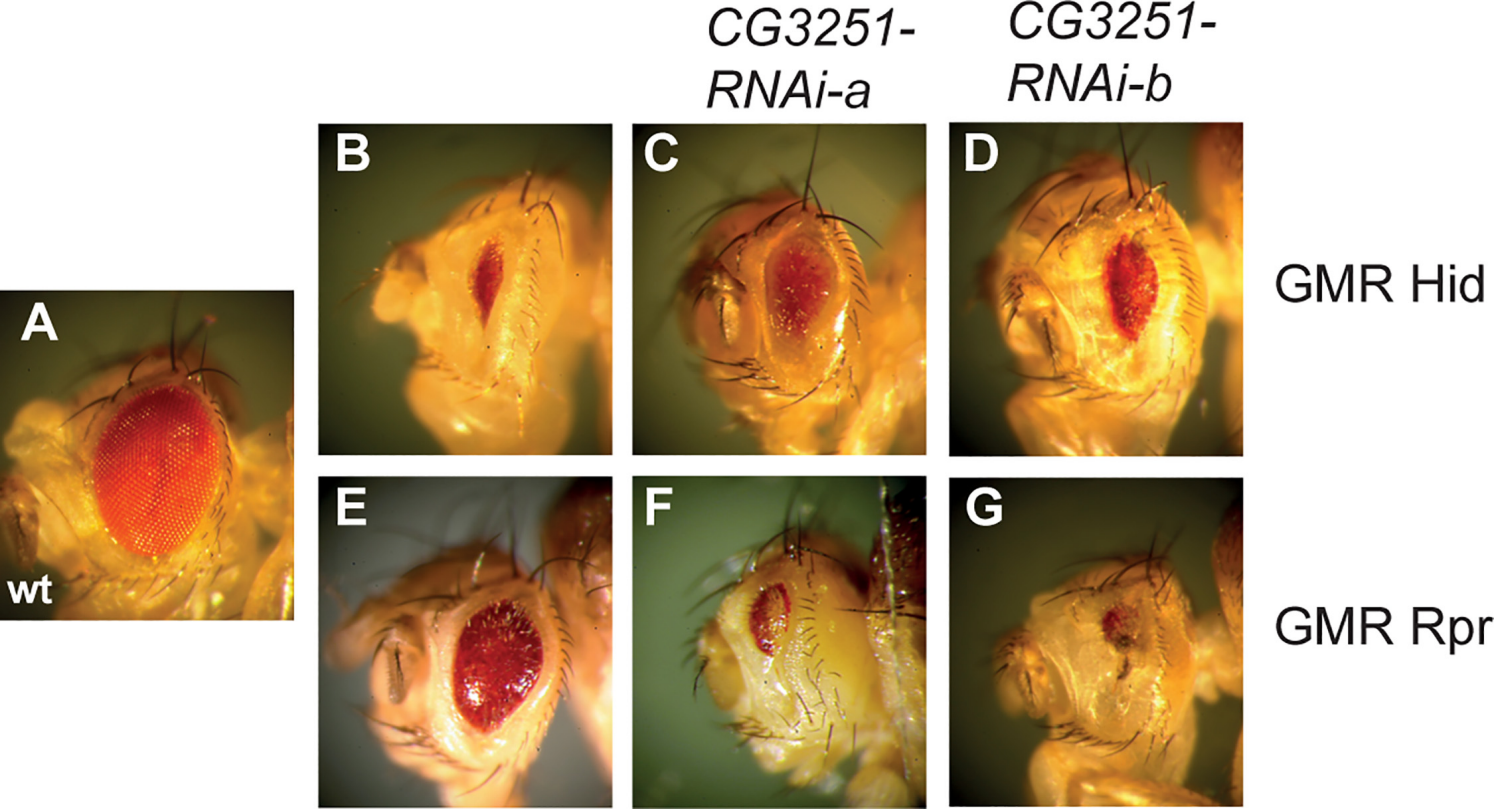
Knock-down of *CG3251* disturbs IAP-antagonist induced apoptosis in the *Drosophila* eye. An intact wild type *Drosophila* eye is shown in **(A)**. Rpr and Hid as well as RNAi constructs were expressed using the UAS-*Gal4* system with the *glass multimer reporter* (*GMR*)-*Gal4* driver, which drives expression mainly in the developing eye and leads to a small, rough eye phenotype due to excessive apoptosis (**B,E**). Knock-down of *CG3251* suppressed Hid-induced cell death in the eye (**C,D**) and enhanced Rpr-induced cell death (**F,G**). Genotypes are: (**B**) *GMR*-*hid*, *GMR*-*Gal4*/*cyo* (**C**) *GMR*-*hid*, *GMR*-*Gal4*/*CG3251-RNAi-a* (**D**) *GMR*-*hid*, *GMR*-*Gal4*/*CG3251-RNAi-b* (**E**) *GMR*-*rpr*, *GMR*-*Gal4*/*TM6b*,*Tb* **(F)** *CG3251-RNAi-a*/*+; GMR*-*rpr*, *GMR*-*Gal4*/*+* **(G)** *CG3251-RNAi-b*/*+; GMR*-*rpr*, *GMR*-*Gal4*/*+*.

No biological function of *CG3251* has been described so far and our results suggest an ubiquitin-independent role. In an independent approach, we have identified *CG3251* as a potential modulator of apoptosis in a well-established model system in the fly eye. Ectopic expression of the pro-apoptotic molecules Rpr and Hid in the developing *Drosophila* eye leads to a small eye phenotype due to apoptotic cell death [20–22], (Fig 5B and 5E, compare to wild type eye in Fig 5A). This phenotype can be enhanced or suppressed by modulators of the apoptotic cascade. RNAi-mediated down-regulation of *CG3251* in two independent RNAi fly lines suppressed the Hid-induced small eye phenotype (Fig 5C and 5D) and enhanced Rpr-induced cell death severely (Fig 5F and 5G). While the mechanism by which CG3251 influences apoptotic signaling remains to be elucidated, our results suggest that despite loss of its DUB activity, CG3251 is required to regulate physiological processes, presumably in a ubiquitin-independent manner.

## Discussion

DUBs of the OTU family normally rely on a cysteine residue in the catalytic triad to process ubiquitin chains and often display specificity for certain linkage types. The *Drosophila melanogaster* proteins CG3251 and Otu contain a serine in this position, while the other two residues of the catalytic triad (aspartate and histidine) are well conserved. Our biochemical analysis indicates that both proteins are not able to process ubiquitin chains *in vitro*. Surprisingly, re-mutation (S>C) in the catalytic Cys-loop did not render the enzymes active, suggesting that in these two proteins further essential features required for the cleavage of ubiquitin chains have been lost or modified. Closer sequence examination of the three loops involved in ubiquitin chain orientation and cleavage, Cys-loop, His-loop and V(ariable)-loop [6], indicates few further amino acid substitution in CG3251 and Otu. While it is difficult to assess functional impact solely based on amino acid sequence, a striking difference in the CG3251 His-loop is the presence of a methionine (M) as the first amino acid, while in other (active) OTU DUBs, this position is taken by an aromatic residue, which contacted the C-terminus of the distal ubiquitin in a model substrate [6]. Future work will have to assess further residues that are essential for activity of CG3251 and Otu.

Interestingly, multiple independent mutations (Cys to Ser) in genes of the OTUD4 family seem to have occurred in different species. Assuming that a Cys to Ser change alone is sufficient to abolish DUB activity, this raises the question why activity of OTUD4 family DUBs is dispensable in many species and whether those proteins that retain an intact catalytic triad actually fulfill DUB function. Both human DUBs of this family, OTUD4 and ALG13, contain an intact catalytic triad. However, only OTUD4 has been shown to possess catalytic activity *in vitro* [6, 16]. A recent publication suggested a role for OTUD4 in DNA alkylation repair [16]. OTUD4 regulated stability of the demethylase ALKBH3 but did not require its own catalytic activity to do so. Instead, it seemed to act as a scaffold protein, recruiting two further DUBs, USP7 and USP9x, which then de-ubiquitylated ALKBH3. These findings support the possibility that catalytic activity of OTUD4-family DUBs is dispensable.

A handful of human DUBs from the USP family with presumably inactivating changes in the catalytic triad have been reported [23, 24]. A likely scenario for inactive DUBs is a function as ubiquitin receptor by binding to ubiquitin chains without cleaving them. However, we were not able to detect significant binding of ubiquitin chains to CG3251 *in vitro* (data not shown), supporting the notion that additional changes in the OTU domain make this DUB inactive for ubiquitin processing and also for ubiquitin binding. A further possible function of CG3251 and Otu could be protease activity towards ubiquitin-like (UBL) molecules. Cross-specificity of DUBs for UBLs has been described, for example for viral OTU proteases and several human USP DUBs that are able to remove ubiquitin as well as ISG15 from cellular proteins [25–27]. In contrast, cross reactivity of DUBs for Nedd8 or SUMO has not been confirmed [27] and these modifiers are usually processed by ULP (UBL-specific protease)-/SENP (sentrin-specific protease)-type proteases, which carry distinct protease domains [28, 29]. While *Drosophila* does not express an ISG15 homologue, we have tested activity of CG3251 and CG3251 S40C against fluorogenic substrates, Nedd8-AMC and SUMO2-AMC, but no processing could be detected (data not shown). Together with the finding that neither CG3251 nor CG3251 S40C were able to cleave a wide panel of peptide substrates for cysteine and serine proteases (data not shown), our data suggest that CG3251 does not harbor any kind of proteolytic activity.

This raises the question about the cellular functions of CG3251 and Otu as inactive OTU-containing proteins. While to our knowledge no reports exist regarding a biological role of CG3251, our RNAi experiments indicate that CG3251 might act in the regulation of apoptosis, presumably in an ubiquitin-independent manner.

The role of Otu in *Drosophila* oogenesis has been widely studied [8]. Several alleles with different mutations with defects in the oocytes have been described [30–32] and also the effect of C-terminal truncations has been tested systematically [33]. While it seems clear that the NH_2_-terminal half (aa 1–423, containing OTU- and TUDOR domain) is required for Otu function in germ cell proliferation and egg chamber differentiation, neither the role of the OTU domain nor its catalytic activity has been addressed in this context. Our results suggest that Otu regulates oogenesis in a DUB-independent manner.

In summary, our analysis indicates that the two *Drosophila melanogaster* members of the OTUD4-family have lost catalytic activity by a Cys-to-Ser replacement in the catalytic triad and one or several further inactivating events. The loss of the catalytic Cys in this protein family seems to have happened frequently in the *Drosophila* genus but occasionally also in other insects or fish. Future studies will have to address whether DUB activity of Cys-containing OTUD4-family members, for example of human OTUD4, is required *in vivo*.

## Material and Methods

### Expression constructs

The expression construct for CG3251 was cloned from S2 cell cDNA into pMT 3xHA vector (modified from Invitrogen) or pGEX-4T3 (GE Healthcare) for expression in bacteria. pMT 3xHA-lacZ was kindly provided by T.Tenev (ICR London). Otu^1-149^ was derived from cDNA clone FI 18803 (DGRC) and cloned into pGEX-6P1. Constructs were verified by DNA sequencing. Point mutants of CG3251 and Otu were created by standard site-directed mutagenesis.

### Recombinant proteins

GST-CG3251, GST-Otu and GST were expressed in *E*. *coli* (BL-21). Expression was induced with 1 mM IPTG (18°C, o/n) and proteins were purified by GST affinity purification. The GST-tag of GST-CG3251 was cleaved off with Thrombin (GE Healthcare) or GST-fusion proteins eluted with Glutathione. His-USP2_CD_ was purchased from R&D systems.

### Transfection of Drosophila cells

Schneider 2 (S2) cells [34] (kindly provided by Pascal Meier, London) were cultured in Schneider’s Drosophila Medium (Gibco) complemented with 10% FBS (Biochrom) and 1% Penicillin/Streptomycin (Invitrogen) at 25°C. Cells were transfected using Effectene transfection reagent (QIAGEN) according to manufacturer’s instructions. Expression of pMT constructs was induced with 350 μM CuSO_4_ over night. Cells were lysed in RIPA buffer (50 mM Tris, pH8, 150 mM NaCl, 1% NP-40, 0.5% Desoxycholate, 0.1% SDS, 10% Glycerol, 1mM DTT, 1 mM Pefabloc).

### DUB activity assays

DUB activity was tested using 1 μg recombinant protein (CG3251, GST or His-USP2_CD_), which was pre-incubated with 10 mM DTT in reaction buffer for 10 min. DUB reaction was carried out with 0.5 μg K48- or K63-ubiquitin chains or 0.25 μg di-ubiquitin in a total volume of 20 μl DUB reaction buffer (50 mM Tris, pH7.5, 1 mM EDTA, 1 mM DTT) at RT over night. The reaction was stopped by addition of SDS sample buffer and heating of samples at 65°C for 5 min.

Alternatively, HA-tagged CG3251 or lacZ as control were purified from S2 cell lysate. For this, α-HA coupled agarose beads (Sigma-Aldrich) were incubated with 100 μg BSA in 250 μl RIPA buffer for 1 h at 4°C and then added to S2 cell lysate to purify HA-CG3251 or HA-lacZ. Beads were washed four times with RIPA buffer and once with DUB reaction buffer. Cleavage reaction was carried out as described above.

### Antibodies

Primary antibodies used for Western Blots were: α-ubiquitin (07–375, Millipore), α-HA 3fl0 (Roche), α-GST (Invitrogen). Secondary HRP-coupled antibodies were from Santa Cruz Biotechnology.

### Drosophila genetics

*GMR*-*rpr*, *GMR*-*GAL4* and *GMR*-*hid*, *GMR*-Gal4 and *Ey*-Gal4 lines [35, 36] were kindly provided by Pascal Meier (ICR London). RNAi lines for *CG3251* were obtained from VDRC: *CG3251-*IR-a = 34574, *CG3251-*IR-b = 100532. All crosses were performed at 25°C and fly eyes photographed using a stereo microscope (Leica) equipped with a Plan Apo 1.0X lense, a digital camera and image capture software (both Nikon).
